# Application of ^1^H and ^13^C NMR Fingerprinting as a Tool for the Authentication of Maltese Extra Virgin Olive Oil

**DOI:** 10.3390/foods9060689

**Published:** 2020-05-26

**Authors:** Frederick Lia, Benjamin Vella, Marion Zammit Mangion, Claude Farrugia

**Affiliations:** 1 Department of Chemistry, University of Malta, 2080 MSD Msida, Malta; ben.vella.16@um.edu.mt (B.V.); claude.farrugia@um.edu.mt (C.F.); 2 Department of Physiology and Biochemistry, University of Malta, 2080 MSD Msida, Malta; marion.zammit-mangion@um.edu.mt

**Keywords:** extra virgin olive oil, authentication, chemometrics, proton NMR, carbon NMR, machine learning, artificial neural networks, PLS-DA

## Abstract

The application of ^1^H and ^13^C nuclear magnetic resonance (NMR) in conjunction with chemometric methods was applied for the discrimination and authentication of Maltese extra virgin olive oils (EVOOs). A total of 65 extra virgin olive oil samples, consisting of 30 Maltese and 35 foreign samples, were collected and analysed over four harvest seasons between 2013 and 2016. A preliminary examination of ^1^H NMR spectra using unsupervised principle component analysis (PCA) models revealed no significant clustering reflecting the geographical origin. In comparison, PCA carried out on ^13^C NMR spectra revealed clustering approximating the geographical origin. The application of supervised methods, namely partial least squares discriminate analysis (PLS-DA) and artificial neural network (ANN), on ^1^H and ^13^C NMR spectra proved to be effective in discriminating Maltese and non-Maltese EVOO samples. The application of variable selection methods significantly increased the effectiveness of the different classification models. The application of ^13^C NMR was found to be more effective in the discrimination of Maltese EVOOs when compared to ^1^H NMR. Furthermore, results showed that different ^1^H NMR pulse methods can greatly affect the discrimination of EVOOs. In the case of ^1^H NMR, the Nuclear Overhauser Effect (NOESY) pulse sequence was more informative when compared to the zg30 pulse sequence, since the latter required extensive spectral manipulation for the models to reach a satisfactory level of discrimination.

## 1. Introduction

Several international organisations, including the European Union through its directives (EC No. 2568/1991 and its amendments) [1] and the International Olive Oil Council (COI/T.15/NC No. 3/ Rev 6) [2], have been at the vanguard in the development of methods and establishing limits for physicochemical parameters of extra virgin olive oil (EVOO) to protect against frauds. The typical approach relies on comparison of the chemical composition with official limits, as it is expected that the presence of adulterants will modify the concentration of these constituents. Nonetheless, this procedure may be inadequate, especially for oils which are classified as ‘virgin’ but do not conform to official limits of certain constituents due to local climatic or soil peculiarities [3]. Furthermore, these methodologies do not address the problem of geographical traceability and tend to be rather time-consuming with a very low throughput.

During the last decade, nuclear magnetic resonance (NMR) spectroscopy has been shown to be highly effective in the study of oils of vegetable origin [4,5,6,7,8]. In 1999, Vlahov [9] proposed NMR spectroscopy as a new analytical tool to compete with the existing methods for studying olive oil chemistry. Among the vast applications of NMR spectroscopy to the study of EVOO, target analysis of triacylglycerides, fatty acids, unsaturated fatty chains for quantification, seed oil adulteration, and degradation of EVOOs encompass some of the techniques that could employ the use of NMR. Furthermore, NMR spectroscopy could also be extended to the study of minor constituents including phenolic compounds, sterols, and phospholipids for both detection and quantification of markers for geographical origin and cultivar information. The main methods used in NMR include ^1^H and ^13^C NMR spectroscopy as reviewed by a number of authors [9,10,11,12,13,14] together with ^31^P NMR as employed by Spyros and Dais [15]. Apart from target-based analytical approaches, NMR metabolic fingerprinting [16,17,18] employs the use of whole NMR spectral data to classify a relevant number of samples according to their origin, harvest, and age. In most cases, fingerprinting analysis is used in conjunction with sophisticated statistical and mathematical procedures.

^1^H NMR has been much more widely used in the field of olive oil chemistry than ^13^C NMR. While requiring more concentrated samples than ^1^H NMR, ^13^C NMR spectra have a much wider radiofrequency range. Coupled with proton decoupling techniques, this leads to sharp spectra which rarely have overlapping carbon peaks, allowing easy detection of impurities and making the peaks readily interpretable. The main disadvantage in ^13^C NMR is the long acquisition times which reduces the sample throughput, unlike ^1^H NMR which takes around 10 min for the entire run to be completed. Preedy and Watson [19] suggest that each type of NMR spectroscopy could be used for a different type of analysis into the composition of olive oil—the ^13^C technique is useful in characterisation of the genotype of the oil, while the ^1^H NMR technique is more suited to geographical characterisation of the oils.

The combination of ^1^H and ^13^C NMR fingerprinting with multivariate analysis provides a promising approach to studying the profile of olive oils in relation to their geographical origin. The Maltese olive oil industry makes an interesting case, as the industry has only recently been regenerated using an indigenous olive stock. Considering the small state of the market, mislabeled EVOO originating from other countries sold as Maltese EVOO could severely impede the growth of the industry, with severe negative economic repercussions. Recent studies have shown that Maltese EVOOs have a significantly different phenolic composition and mineral composition [20,21,22]. In this study, a variety of olive oils selected from different areas around the Maltese islands and countries around the Mediterranean were studied. No data is present in the literature regarding the use of ^13^C and ^1^H NMR for the authentication of Maltese EVOOs. The aim of this study was to explore the use of ^13^C NMR and ^1^H NMR (specifically ^1^H zg30 and ^1^H NOESY), in conjunction with chemometrics in order to differentiate the Maltese EVOOs from other EVOOs derived from other countries within the Mediterranean region, thus developing an easy and cost-saving verification method for the origin of EVOOs from the Maltese islands ensuring olive oil chain sustainability.

## 2. Materials and Methods

### 2.1. Sample Preparation

For this preliminary study, a total of 65 extra virgin olive oil samples were collected from the Maltese islands over four harvest seasons from 2013–2016 and from other neighboring Mediterranean countries. The cultivars used in this study and their country of origin can be seen in Table S1. The samples were all taken from different oil producers to cover a representative sample of the Maltese islands in terms of pedological and microclimatic conditions, whilst also accounting for manufacturing techniques and the different presses employed. Foreign olive oils obtained were bought with a protected designation of origin in order to ensure traceability of the product. All the samples were stored at 4 °C in the absence of light prior to the analysis. The samples were preheated to 35 °C in a water bath for 1 h and mixed to ensure homogeneity. For ^1^H NMR, 20 µL of the EVOO were placed in 5 mm NMR tubes and dissolved in 700 µL of deuterated chloroform, followed by the addition of 20 µL of deuterated DMSO and vortex mixing for 20 s. For ^13^C NMR, 440 µL of sample was dissolved in 420 µL of deuterated chloroform without the addition of DMSO [23].

### 2.2. ^1^H and ^13^C NMR Spectra Acquisition

The analysis was performed on a model AVANCE III 500 MHz NMR spectrometer equipped with a 5 mm ^1^H/D-BB probehead with z-gradient, automated tuning and matching accessory, and a BTO-2000 accessory for temperature control (Bruker BioSpin GmbH, Rheinstetten, Germany). Samples were measured at 300.0 K after a 5 min resting period for temperature equilibration. NMR spectra were acquired using Topspin 3.5 (Bruker). Automated tuning and matching, locking and shimming using the standard Bruker routines, ATMA (automatic tuning and matching in automatic mode), LOCK (frequency-field lock to offset the effect of the natural drift of the NMR’s magnetic field B0) and TopShim, were used to optimise the NMR conditions. Samples were analysed using the zgpg30 pulse method for ^13^C NMR, while the zg30 and NOESY 1D noesypr1d NMR pulse sequence using a standard presaturation were used for ^1^H NMR. Every extract sample was run twice with a ^1^H NMR standard single pulse experiment zg30 for 100 scans. The samples were run twice automatically under the control of ICON-NMR. Each run had two prior dummy scans, resulting in 65,536 data points with a resolution of 0.305 Hz acquired with an acquisition time and a relaxation delay time of 3.27 and 4 s, respectively. The 90° flip angle for free induction decay was adjusted to 10 µs. In the case of one-dimensional Nuclear Overhauser Effect spectroscopy, 100 scans were acquired, each run having two dummy scans, which resulted in 32,768 data points with a resolution of 0.489 Hz, acquired with an acquisition time and relaxation time of ~2.04 and 4 s, respectively. In the case of ^13^C NMR, 250 scans were recorded for each sample, with an acquisition time of 21 s to allow sufficient time for complete relaxation of ^13^C nuclei between scans. The acquisition delay was set at 2 s. The receiver gain was set at 203, and the temperature was locked at 298.0 K by means of a BTO-2000 accessory. Broadband 1 H decoupling techniques were employed. The above parameters and settings could run samples with a turnover time of 1 h and 40 min each, excluding an initial 5-min temperature equilibration period.

Prior to Fourier transformation, the free induction decays (FIDs) were zero-filled to 64 k and a 0.3 Hz line-broadening factor was applied. The chemical shifts are expressed in d scale (ppm), referenced to the residual signal of chloroform. For ^1^H NMR, this was found at 7.24 ppm [21] whilst for ^13^C NMR, this was found as a triplet centerd around 77.01 ppm [22]. The corrected spectra were exported as ASCII files from Topspin 3.5 (TopSpin™ version 5, Bruker, Billerica, MA, USA) and imported directly into The Unscrambler X 10.3 (CAMO Software, Oslo, Norway) for all subsequent mathematical data processing. Each spectrum was automatically binned by the software into 32,768 buckets, each bucket being 0.0072223 ppm wide. The signal-to-noise ratio was calculated using the peak at 172.8 ppm for ^13^C NMR corresponding to C1 of the glycerol chain, which resulted in a signal-to-noise ratio of 520:1. For ^1^H NMR, the signal-to-noise ratio was calculated using the peak at 9.70 ppm, corresponding to the aldehyde proton in hexanal, and a signal-to-noise ratio of 1.26:1 and 1.46:1 was obtained for zg30 and NOESY pulse sequences, respectively.

The spectrum obtained was subjected to different spectroscopic signal processing techniques, which were evaluated and compared. The spectra were normalised, a transformation that put all spectra on the same scale, thus eliminating the fluctuations in intensities between spectra arising from slightly different sample concentrations. Both peak normalisation and area normalisation were carried out separately on the baseline corrected spectrum. Normalisation was followed by detrending and deresolving procedures. The detrend transformation removes the effects of nonlinear trends, showing only the absolute changes in values across spectra by removing the least-squares line of best fit from the data, thus focusing only on fluctuations between data. Deresolve is a noise-reducing transformation that operates by artificially lowering the resolution of the spectra. Other treatments applied to the baseline corrected spectrum include multiplicative and orthogonal scatter corrections (MSC and OSC), and standard normal variate (SNV). MSC was corrected for scaling effects by performing a regression of a spectrum against a reference spectrum, thereby correcting the spectrum using the slope of the fit was obtained from the regression. OSC removes variance from the factors that is not related to the response, by finding directions in X that describe large variances while being orthogonal to Y and subtracting them from the data. The SNV transformation works similarly to MSC, however, it standardises each spectrum using data from the spectrum itself rather than data averaged from all the spectra. A number of derivatising procedures (1st and 2nd derivatives, Savitzky-Golay) were also carried out. The 1st derivative removes baseline effects while the 2nd derivative also removes the slope of the spectrum by measuring the change in slope, thereby sharpening spectral features. The Savitzky-Golay derivative fits a low-degree polynomial to adjacent points in a spectrum, thereby smoothing the spectrum while minimally affecting the signal-to-noise ratio.

### 2.3. Data Analysis

A principle component analysis (PCA) was carried out using Unscrambler X 10.3 in order to identify any gross outliers and determine any preliminary clustering reflecting the geographical origin. An inspection of the PCA loadings was carried out in order to determine whether the loadings had a spectral shape indicating that observed variation was due to the NMR spectra and not due to noise. PCA was carried out on all treated spectra to reduce all the spectral information down to seven principal components (PCs), which retained the information of the original dataset. The first PC accounted for most of the variation in the dataset, with successive principal components accounting for decreasing amounts of the variation. The resulting PC-1 vs. PC-2 plots could be examined for any clustering that might arise from each spectral pretreatment. Similarly, to PLS, PCA generates loading plots which indicate those x-values which are most responsible for the variability between the different spectra. The loading plots for the first two principal components (which explain most of the variability in the dataset) were used to determine which ppm values had the largest influence on the separation of PC-1 and PC-2. Following a PCA, supervised chemometric methods were carried out using JMP^®^, Version 10 (SAS Institute Inc., Cary, NC, USA), including the partial least squares discriminate analysis (PLS-DA). The whole dataset was split into two sets, termed the training and test sets (the former to build the model, the latter to validate it). In order to preserve the diversity in the training and test sets and to account for the fact that different pretreatments had to be tested, a unique sample splitting scheme was used.

In order to determine the suitability of the whole NMR spectra for discrimination of EVOOs of Maltese origin, an artificial neural network (ANN) analysis was carried out. The main advantage of a neural network model is that it can efficiently model different response surfaces due to its nonlinearity, allowing a better fit to the data given enough hidden nodes and layers, providing an accurate prediction for many kinds of data. Unlike other modeling and discriminate methods (PLS) the main disadvantage of a neural network model is that the results are not easily interpretable, due to the presence of several intermediate hidden layers. In this experiment, 25 iterations were carried out using a TanH activation function as the standard neuron activation function in JMP software. In the case of ANN, three different cross-validation techniques were employed in order to prevent model overfitting; the k-fold (CV-10), hold back (33.3%), and excluded rows (Venetian blinds). Thirty-three percent of the samples were held back from the model during holdback validation, which operates by randomly splitting the dataset into training and validation sets. Thirty-three percent of the data was thus ‘held back’ to form the validation set. Excluded rows holdback uses those rows that were excluded by the Venetian blinds method as the validation set. K-fold validation divides the dataset into ‘k’ number of subsets where each subset contains a fraction ‘1/k’ of the data. Each of these sets is used to validate the model thereby fitting ‘k’ number of models. The best fitting model is presented as the final output. In this study, K-fold validation was carried out using 5 k-folds.

## 3. Results and Discussion

### 3.1. Geographical Classification of EVOO Using NMR Spectroscopy

**Figure 1:**
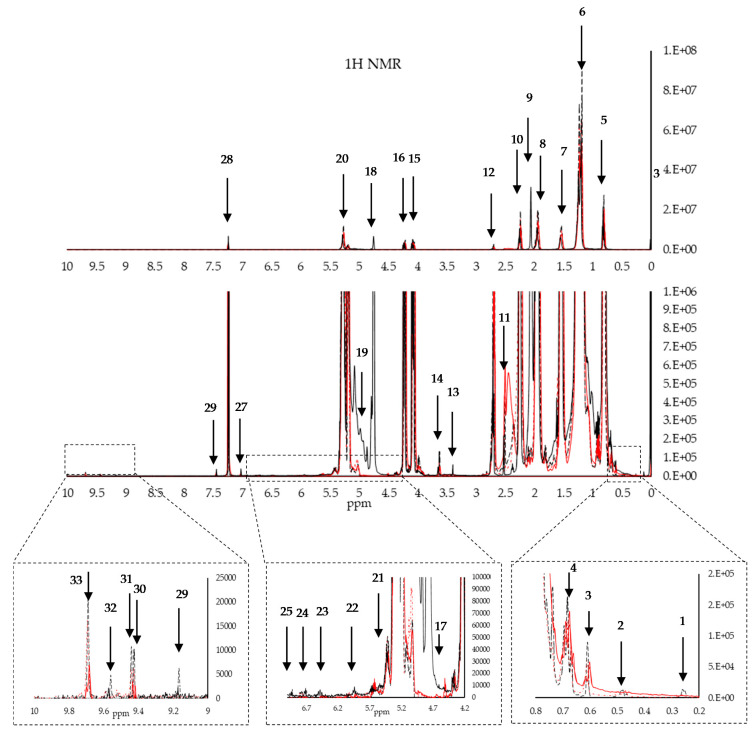
The major peaks of interest obtained from the nuclear magnetic resonance (NMR) of extra virgin olive oils (EVOOs) using the zg30 pulse sequence (black) and NOESY pulse sequence (red).

**Figure 2:**
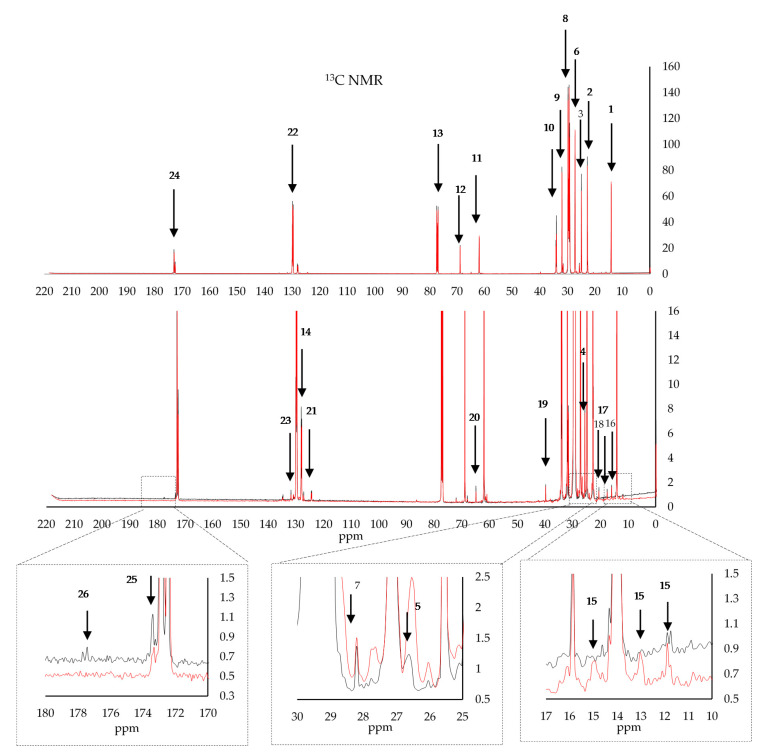
The major peaks of interest obtained using the ^13^C NMR of EVOOs (black line Maltese EVOOs, red line non-Maltese EVOOs).

Figure 1 and Table 1 show ^1^H NMR signals of the major and some minor compounds together with their chemical shifts and their assignments to protons of the different functional groups [10,24,25,26,27,28]. Figure 2 and Table 2 show the major peaks obtained using ^13^C NMR and identified using the literature [10,13,14,17,27,28,29,30,31,32,33].

Whilst the chemical shifts of the major constituents are well known and easily identified, the ^1^H and ^13^C signals of the minor oil components are only observed when their signals do not overlap with those of the main components, and when their concentrations are high enough to be detected [11]. Minor constituents which are expected to yield NMR signals include mono- and diglycerides, sterols, tocopherols, aliphatic alcohols, hydrocarbons, fatty acids, pigments, and phenolic compounds [32]. Figure 1 shows the most common ^1^H NMR signals of the major and some minor compounds together with their chemical shifts and their assignments to protons of the different functional groups. The main identified compounds include; cycloartenol at 0.29 and 0.54 ppm, β-sitosterol at 0.62, 0.67 ppm, stigmasterol at 0.69 ppm, wax at 0.98 ppm, squalene at 1.66 ppm, sn-1,2 diglyceryl group protons at 3.71 and 5.28 ppm, and two unknown terpenes at 4.53, 4.65, and 4.95 ppm, hexanal at 9.7 ppm, and phenolic protons at, 6.95, and 6.72 ppm. These compounds have already been observed and identified by other authors [10,11,14,17,30]. In the case of ^13^C NMR, the minor constituents observed were restricted to chemical shifts corresponding to squalene, with a shouldering peak at 26.6 ppm and another minor peak at 28.2 ppm attributed to the allylic methylene group [26].

The discriminatory models for the traceability of EVOOs from the Maltese islands coupled ^1^H and ^13^C NMR spectroscopy with chemometrics. In order to overcome the instrumental limitation and to account for scattering and other minor variations which would hinder the performance of the classification model, different kinds of spectral pretreatments were tested and compared. A total of 10 spectral pretreatment methods were used. In each case, after pretreatment, a PCA was carried in order to dimensionally reduce the number of variables into a small set of principal components whilst retaining all the information of the larger set. PCA enabled the preliminary identification of which pretreatment offered the highest variability and possible sample grouping based on the geographical origin but also enabled the identification of outliers and noise modeling.

**Figure 3:**
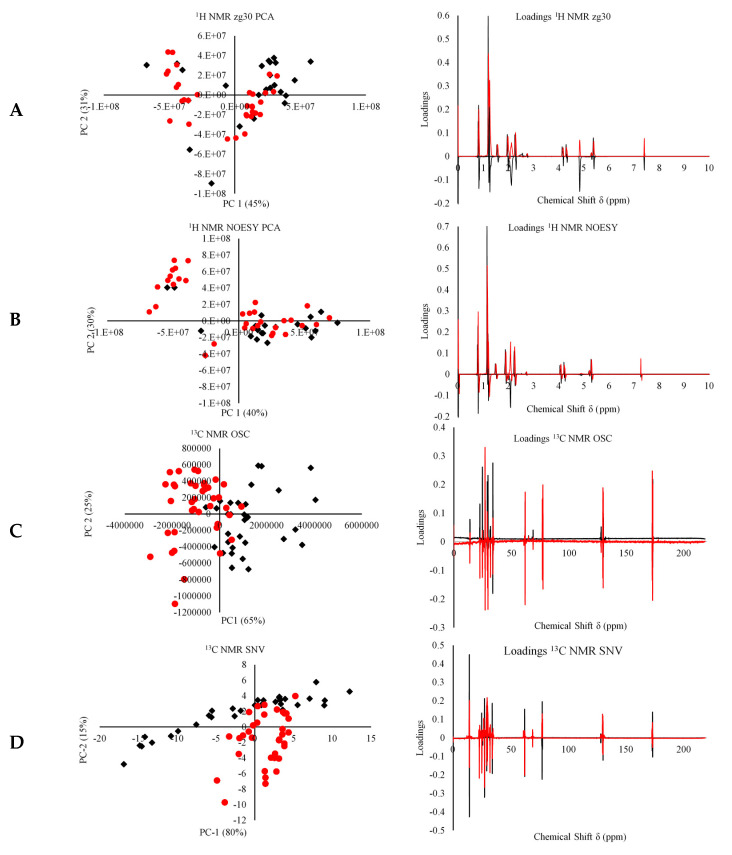
The principle component analysis (PCA) biplots (black boxes = Maltese red dots = non-Maltese) and loading plots for PC1 (black line) and PC2 (red line) for the untreated raw data for the zg30 (**A**) NOESY (**B**), ^13^C NMR orthogonal scatter corrections (OSC) (**C**), and ^13^C NMR standard normal variate (SNV) (**D**) spectra.

Figure 3 shows some of the different forms of spectral pretreatments employed and the corresponding PCA plot for the first two principal components. In the case of ^1^H NMR, although clustering was observed in most of the spectral pretreatments, it did not fully discriminate the EVOOs of Maltese origin from those obtained from other Mediterranean countries. Only a weak clustering resembling the geographical origin was observed by using PCA. For ^1^H NMR, the raw data was presented in Figure 3 as these were seen as the most representative data for highlighting clustering in PCA. Other spectral transformations can be viewed in the Supplementary Materials Figures S1–S3. In the case of ^13^C NMR, the clustering obtained using OSC and SNV spectral transformations highly resembled the geographical origin of EVOO.

Inspection of the PC loadings revealed a spectral form, which suggests that the variation observed was due to the actual NMR spectra and not due to noise. In the case of zg30, it was observed that the chemical shifts observed at 0.8 and 1.2–1.25 ppm and 0.5–1.25 ppm for the NOESY experiment seem to have a larger influence on the first and second principal component separation. These observations suggest that the phytosterol content, namely β-sitosterol, campesterol, cycloartenol together with 1-eicosanol and α-tocopherol, which show chemical shifts between 0.5–1.25 ppm, have a greater influence on the variation observed along the first two principal components. In the case of zg30, other peaks observed in the 4.7–4.9 ppm range also seem to be influential, especially in the 1st PC, these peaks correspond to terpenic compounds present in EVOOs. Alonso-Salces et al., [17,30] identified three peaks at 4.57, 4.65, and 4.70 ppm, which were attributed to unknown terpenes during their study on the unsaponifiable fraction of EVOOs. For ^13^C NMR, inspection of the PC loading plots corresponding to the previously identified chemical shifts were found to offer the most variation, with the peak at 14 ppm assigned to the terminal –CH_3_ of all acyl chains explaining most of the variation in the SNV spectra.

### 3.2. Application of PLS-DA for the Discrimination of Maltese EVOOs

The Maltese and the non-Maltese samples were grouped in ascending order so that the first 30 samples would represent Maltese EVOOs whilst the rest corresponded to non-Maltese EVOOs. A Venetian blinds cross-validation method was then employed, which selected every sth sample from the data by making data splits such that all samples are left out exactly once (s = 5). This sampling method excluded 20% of the dataset so that they would be retained as the testing set. The remaining 80% of the dataset was used to build the training set. In the case of PLS-DA, an inspection of the variable importance plot (VIP) scores was carried out. Variables having a smaller VIP than 0.8 were removed, and an adjusted PLS model was built after the removal of these variables. The goodness of fit of the adjusted model was evaluated and compared to the original model. Table 3 shows the accuracy (% correct classification during training) and the precision (% correct classification during testing) obtained on using different spectral pretreatments for the two NMR methods. For the zg30 NMR spectra obtained after deresolve, SNV and quantile normalisation showed the best model performance with a % accuracy ranging from 93.1–87.9% and % predictability ranging from 72.7–81.8%, whilst for the NOESY experiment, spectra treated using normalisation and Savitzky-Golay showed the best performance with an accuracy of 94.8% and predictability of 90.9%. In the case of the zg30 experiment, all the spectral pretreatments showed an improvement in the % predictability when compared to the raw data, whilst in the NOESY experiment, spectra treated using SNV and detrending functions showed a lower % predictability and % accuracy when compared to actual nonpretreated raw data. This observation suggests that, in the case of NOESY, the signal suppression of the major peaks improves the signal to noise ratio, and the resulting spectra can be used without the need of extensive pretreatments. Results obtained by Longobardi et al., [18] showed that the presaturation of the dominating lipid signals resulted in an increased receiver gain which in turn resulted in a signal-to-noise gain close to 10 compared to the zg30 spectra. In the case of ^13^C NMR, higher rates of accuracy and predictability were observed when compared to ^1^H NMR methods with a % predictability ranging from 66.7–100%, with OSC reaching 100% correct classification in both the training and validation sets. The higher rates of predictability of ^13^C NMR spectra were attributed to a higher signal-to-noise ratio, less coupling interactions resulting in a cleaner signal, proof of this is the % predictability of the raw untreated ^13^C spectra with respect to ^1^H spectra.

The next step was to build another PLS model, this time using only variables which had a VIP score > 0.8. Table 3 also shows the results obtained by using the adjusted PLS model for ^13^C and ^1^H NMR. An improvement in the overall % accuracy and predictability of the model. Furthermore, the models obtained using only VIP > 0.8 variables showed an increase in both %X and %Y explained, and a higher % accuracy and % precision indicating enhanced model performance. In the case of the zg30 experiment, it was found that normalised spectra and Savitzky-Golay derived spectra had the optimal performance, whilst detrended and SNV spectra had optimal performance when the whole data set was used. In the case of the NOESY experiment, the models obtained using VIP > 0.8 showed an increase in the performance when compared to those obtained with whole data. 

These observations indicate that different spectral pretreatments are affected differently to variable selection techniques since each one of them attempts to maximise spectral variations and corrections, therefore, removal of a small number of predictors can have a devastating effect on the model performance. In the case of ^13^C NMR, variable selection greatly improved the discrimination with most of the pretreated spectra reaching 100% accuracy and predictability. The noticeable increase in the model performance has been attributed to the removal of redundant variables which correct for overfitting by excluding noise variables from the data, therefore, preventing them from affecting the model. Reducing the number of variables around which the model is built also increases the model’s reliability.

### 3.3. Whole ^1^H and ^13^C-NMR Modeling Using Feed-Forward Predictive Artificial Neural Networks

The use of feed-forward predictive neural networks on the NMR data as a method for classification was assessed using three different forms of validation, namely 33.3% data holdback, CV-10 k-fold, and excluded row validation. Since ANNs are more powerful than any other classification method in terms of their flexibility and noise insensitivity, the algorithm was fitted on the training set using the entire NMR spectrum without any form of variable selection. Table 4a,b shows % accuracy and % predictability for the different forms of cross-validation carried out on different spectral pretreatments of ^13^C NMR and ^1^H NMR under the zg30 and NOESY NMR spectra. In general, contrary to what was observed in PLS-DA, it was observed that models obtained under ^1^H NMR models had higher rates of classification when compared to ^13^C NMR. Similarly, to what was observed in PLS-DA, raw data derived from the NOESY experiment had a higher model performance throughout the three different validation methods used when compared to the zg30 experiment. With respect to validation it was observed that, irrelevant to the spectrum used, the 33% holdback cross-validation resulted in overfitted models which were identified as spectral transformations that had very good training models but failed to predict new samples with the exception of MSC. In general, the best performing cross-validation method was the excluded row validation followed by k-fold validation. This could be attributed to the fact that these cross-validation methods are not completely random as the 33% holdback. In the case of the excluded row validation the samples were selected in such a way that, the groups contained approximately equal amounts of local and foreign EVOOs in the training stage. Thus, the models obtained where equally capable of recognising and predicting local and foreign EVOOs.

## 4. Conclusions

It was shown that different NMR methods in conjunction with chemometric methods provided a new insight in the identification of Maltese EVOOs. From the preliminary assessment using only unsupervised PCA models, no significant clustering was observed, and this was attributed to the high levels of similarity between the two classes of EVOOs studied, therefore, this method was deemed to be unsatisfactory when it comes to discrimination of geographical origin. The application of supervised methods of classification, namely PLS-DA and ANN, were shown to be highly effective in discriminating local and nonlocal EVOO samples. The use of the variable selection methods significantly increased the effectiveness of PLS-DA models in discriminating Maltese EVOOs. ANN models were also shown to offer similar classification rates to PLS-DA models and thus they corroborate the results obtained. Results showed that different NMR pulse methods can greatly affect the discrimination of EVOOs. The most informative method was ^13^C NMR, which resulted in a cleaner spectrum which was void of coupling, followed by the ^1^H NOESY pulse sequence, in which suppression of strong signals greatly improved the signal-to-noise ratio when compared to the zg30 ^1^H NMR spectra. NMR data acquired using the zg30 pulse sequence required an extensive spectral elaboration in order to obtain a comparable model performance to that of ^1^H NOESY and ^13^C NMR. It was concluded that apart from the initial and running costs of the instrumentation, NMR proved to be a cheap and reliable technique for the discrimination of Maltese EVOOs from non-Maltese EVOOs. Whilst ^13^C NMR was very successful in the discrimination of Maltese EVOOs, the long acquisition time proved to be unsatisfactory for a high throughput analysis and thus it is proposed to be used as a confirmatory method for the identification of origin.

## Figures and Tables

**Table 1:** Chemical shifts and the corresponding chemical functional group observed for ^1^H NMR.

^1^H NMR					
	Chemical Shift	Compound Functional Group		Chemical Shift	Compound Functional Group
1	0.29	-CH_2_-(cyclopropanic ring) cycloartenol	17	4.53	Terpene
2	0.54	-CH_2_-(cyclopropanic ring) cycloartenol	18	4.65	Terpene
3	0.62	-CH_3_(C18-steroid group) β-sitosterol	19	4.95	Terpene
4	0.69	-CH_3_(C18-steroid group) β-sitosterol	20	5.28	>CHOCOR (glyceryl group)
5	0.81	-CH_3_(acyl group)	21	5.55	Unk 2-Tocopherols
6	1.19	-(CH_2_)n-(acyl group)	22	5.91	-CH=CH-CH=CH-(cis, trans conjugated dienediene system)
7	1.54	-OCO-CH_2_-CH_2_-(acyl group)	23	6.56	-CH=CH-CH=CH-(cis, trans conjugated dienediene system)
8	1.95	-CH_2_-CH=CH-(acyl group)	24	6.72	-Ph-H (phenolic ring)
9	2.08	-CH_2_-CH=CH-(acyl group)	25	6.95	-Ph-H (phenolic ring)
10	2.26	CH-CH_2_-CH=(acyl group)	26	7.02	Chloroform ^13^C satellite
11	2.54	CH-CH_2_-CH=(acyl group) satellite	27	7.24	Chloroform
12	2.71	CH-CH_2_-CH=(acyl group)	28	7.44	Chloroform ^13^C satellite
13	3.39	Unk 1-alcohol	29	9.17	Unk 4-hydrocarbon
14	3.71	-CH_2_OCOR (glyceryl group)	30	9.46	Unk 5-hydrocarbon
15	4.10	-CH_2_OCOR (glyceryl group)	31	9.47	Unk 5-hydrocarbon
16	4.22	-CH_2_OCOR (glyceryl group)	32	9.58	Unk 6-hydrocarbon
			33	9.70	Hexanal

**Table 2:** Chemical shifts and the corresponding chemical functional group observed for ^13^C NMR.

^13^C NMR					
	Chemical Shift	Compound Functional Group		Chemical Shift	Compound Functional Group
1	14	C18(ω1) terminal carbon of fatty acyl chain	14	128.01 129.82	C9, C10 oleoyl unsaturated carbons between 2- and 1(3) of glycerol
2	22.64	C17(ω2) penultimate carbons from the fatty acyl chains	15	11.97,13.12,14.95	Unk 1 possibly being attributed to waxes
3	24.74	C3 methylenic group in β position with respect to the carbonylic group	16	15.95	Unk 2 possibly C8′a and C4′a of tocopherols
4	25.53	C11 Linoleyl Linolenyl	17	17.51	Unk 3 possibly C12′a of tocopherols
5	26.61	C8 allylic methylenes of sqaulene	18	20.47	C17(ω2) all acyl chains
6	27.12	C8 allylic carbons of oleoyl chains	19	39.68	Unk 4-C1 of tocopherol
7	28.6	C12 allylic methylenes of sqaulene	20	64.89	Unk 5 possibly C2 of elenolic acid derivative of tyrosol or hydroxytyrosol
8	29.28	C4–C7, C12–C15, C8–C15, C8–C13 methylenic groups in fatty acid central chain	21	124.40	C3′, C7′, C11′ of tocopherols
9	31.88	C16 methylenic acylic chains ω	22	131.70	C9 Linoleyl and linolenyl, C13 Linoleyl
10	33.91	C2, sn-2 acyl chains	23	134.80	C4′, C8′ of tocopherols
11	61.93	CH_2_O-1(3) glycerol carbons of triglycerides	24	172.8	C1, sn-2 2-glycerol chain
12	68.86	CH_2_O-2-glycerol carbon of triglycerides resonates	25	173.2	C1, sn-1,3 1(3) glycerol chain positions
13	77.39	CDCl_3_ Solvent	26	177.92	Unk 6-COOCH_3_ of elenolic acid

**Table 3:** The PLS-DA analysis on both the entire (**a**) ^1^H NMR and (**b**) ^13^C NMR spectra and selected variables having a VIP > 0.8 for the different spectral pretreatments. The results obtained on the training dataset are given in terms of % accuracy of correct classification whilst for the testing data set these are given in terms of % predictability of correct classification.

(a)				
Pretreatment	zg30 ^1^H NMR			
	Whole Spectrum		VIP > 0.8	
	% Accuracy	% Predictability	% Accuracy	% Predictability
Raw	77.59	27.27	82.76	45.45
Normalised	91.38	63.64	94.83	90.91
Q Norm	93.10	72.73	94.83	72.73
Detrend	70.69	36.36	68.97	36.36
Deresolve	87.93	63.64	82.76	45.45
SNV	93.10	81.82	60.34	36.36
MSC	91.38	81.82	67.24	63.64
OSC	72.41	45.45	94.83	72.73
Savitzky-Golay	74.14	54.55	98.28	90.91
1st Derivative	68.97	45.45	94.83	72.73
2nd Derivative	77.59	63.64	93.10	63.64
**Pretreatment**	**NOESY ^1^H NMR**			
	**Whole Spectrum**		**VIP > 0.8**	
	**% Accuracy**	**% Predictability**	**% Accuracy**	**% Predictability**
Raw	82.76	45.45	93.10	75.00
Normalised	94.83	90.91	94.83	83.33
Q Norm	94.83	72.73	96.55	91.67
Detrend	68.97	36.36	74.14	66.67
Deresolve	82.76	45.45	94.83	83.33
SNV	60.34	36.36	70.69	75.00
MSC	67.24	63.64	68.97	75.00
OSC	94.83	72.73	96.55	83.33
Savitzky-Golay	98.28	90.91	89.66	75.00
1st Derivative	94.83	72.73	93.10	91.67
2nd Derivative	93.10	63.64	93.10	91.67
**(b)**				
**Pretreatment**	**^13^** **C NMR**			
	**Whole Spectrum**		**VIP > 0.8**	
	**% Accuracy**	**% Predictability**	**% Accuracy**	**% Predictability**
Raw	100.00	73.33	100.00	80.00
Normalised	100.00	86.68	94.64	100.00
Q Norm	100.00	80.00	100.00	100.00
Detrend	100.00	66.67	100.00	100.00
Deresolve	78.57	73.33	91.07	100.00
SNV	100.00	86.67	100.00	100.00
MSC	100.00	86.67	100.00	100.00
OSC	100.00	100.00	100.00	100.00
Savitzky-Golay	100.00	73.33	100.00	100.00
1st Derivative	100.00	80.00	100.00	100.00
2nd Derivative	100.00	86.67	100.00	100.00

**Table 4:** Application of artificial neural network (ANN) on the (**a**) ^1^H NMR and (**b**) ^13^C NMR data using three forms of cross-validation.

ANN						
Pretreatment	Holdback		CV-10		Excluded Row	
	Training	Validation	Training	Validation	Training	Validation
**(a)**						
zg30 ^1^H NMR						
Raw	81.03	81.82	96.55	81.82	86.21	90.91
Normalised	94.83	81.82	94.83	100	81.03	63.64
Q Norm	98.28	90.91	98.28	90.91	82.76	81.82
Detrend	77.59	54.55	91.38	90.91	75.86	45.45
Deresolve	91.38	90.91	93.1	90.91	79.31	81.82
SNV	96.55	90.91	98.28	100.00	74.14	72.73
MSC	98.28	100.00	96.55	90.91	93.10	90.91
OSC	77.59	36.36	89.66	63.64	84.48	81.82
2nd Derivative	96.55	90.91	98.28	100.00	86.21	45.45
1st Derivative	84.48	90.91	98.28	90.91	81.03	63.64
Savitzky-Golay	91.38	81.82	98.28	90.91	85.00	90.91
NOESY ^1^H NMR						
Raw	93.33	91.67	93.33	91.67	93.33	91.67
Normalised	95.00	91.67	95.00	100.00	93.33	100.00
Q Norm	98.33	100.00	98.33	100.00	93.33	100.00
Detrend	70.00	83.33	96.67	91.67	95.00	83.33
Deresolve	96.67	100.00	96.67	100.00	91.67	83.33
SNV	93.33	91.67	98.33	100.00	98.33	100.00
MSC	93.33	100.00	93.33	83.33	95.00	91.67
OSC	81.67	75.00	91.67	75.00	90.00	91.67
2nd Derivative	96.67	100	98.33	100.00	96.67	91.67
1st Derivative	93.33	91.67	98.33	100.00	93.33	100.00
Savitzky-Golay	88.33	100	98.33	100.00	90.00	91.67
**(b)**						
^13^C NMR						
Raw	83.93	60.00	100.00	80.00	92.62	80.00
Normalised	91.07	46.67	100.00	100.00	100.00	100.00
Q Norm	78.57	80.00	98.21	86.67	98.21	100.00
Detrend	91.07	80.00	100.00	80.00	98.21	86.67
Deresolve	78.57	73.33	100.00	40.00	87.50	86.67
SNV	94.64	86.67	98.21	93.33	92.86	100.00
MSC	80.36	53.33	100.00	73.33	100.00	86.67
OSC	75.00	66.67	75.00	66.67	100.00	73.33
2nd Derivative	85.71	40.00	100.00	66.67	96.43	86.67
1st Derivative	94.64	73.33	100.00	46.67	100.00	73.33
Savitzky-Golay	83.93	40.00	100.00	73.33	83.93	93.33

## Supplementary Materials

The following are available online at https://www.mdpi.com/2304-8158/9/6/689/s1, Table S1: The cultivars used in this study and their country of origin. Figure S1. The principle component analysis and loading plots for different ^13^C NMR spectra. Figure S2. The principle component analysis biplots and loading plots for different ^1^H zg30 NMR spectra. Figure S3. The principle component analysis biplots and loading plots for different ^1^H NOESY NMR spectra.
