# Supplementary material for: Application of 1H and 13C NMR Fingerprinting as a Tool for the Authentication of Maltese Extra Virgin Olive Oil
Source: Foods. 2020 May 26;9(6):689. doi: 10.3390/foods9060689 (PMC7353642; doi:10.3390/foods9060689)
Supplement: Supplementary file 1 [file foods-09-00689-s001.pdf]

Back Matter

Supplementary Material.

Table S1. The cultivars used in this study and their country of origin.

Supplier	Origin	Year	Cultivar
Sample 1	Italy	2014	Nocellara
Sample 2	Italy (Firenze)	2015	Frantoio
Sample 3	Italy	2015	Leccino
Sample 4	Italy	2015	Olivobianco
Sample 5	Italy	2015	Pendolino
Sample 6	Italy (Calabria)	2016	Carolea
Sample 7	Italy (Puglia)	2016	Ogliarola Bio
Sample 8	Italy (Puglia)	2016	Peranzana
Sample 9	Italy	2016	Coratina
Sample 10	Italy	2016	Cerasuola
Sample 11	Italy (Firenze)	2016	Moraiolo
Sample 12	Italy	2016	Leccio del Corno
Sample 13	Italy (Abruzzo)	2016	I-77
Sample 14	Italy	2016	Tortiglione
Sample 15	Italy	2016	Taggiasca
Sample 16	Italy	2016	Gentile di Chieti
Sample 19	Sicily	2015	Tonda Iblea
Sample 20	Sicily	2016	Nocellara Etnea
Sample 21	Sicily	2016	Moresca
Sample 22	Sicily	2016	Biancolilla
Sample 23	Sicily	2016	Nocellara del Belice
Sample 24	Sicily	2016	Tonda Iblea
Sample 25	Sicily	2016	Cerasuola
Sample 26	Spain	2016	Arbequina
Sample 27	Spain	2016	Hojiblanca
Sample 28	Spain	2016	Picual
Sample 29	Spain	2016	Arbequina
Sample 30	France	2015	Béruguette
Sample 31	France	2015	Picholine
Sample 32	France	2015	Grossane
Sample 33	Greece	2016	Koroneiki
Sample 34	Greece	2016	Koroneiki
Sample 37	Malta	2013	Malti
Sample 38	Malta	2014	Bidni
Sample 39	Malta	2014	Bidni
Sample 40	Malta	2014	Malti

Sample 41	Malta	2014	Carolea
Sample 42	Malta	2015	Bajda
Sample 43	Malta	2015	Bidni
Sample 44	Malta	2015	Bidni
Sample 45	Malta	2015	Carolea
Sample 46	Malta	2015	Frantoio
Sample 47	Malta	2015	Malti
Sample 48	Malta	2015	Picholine
Sample 49	Malta	2015	Carolea
Sample 50	Malta	2015	Pendolino
Sample 51	Malta	2016	Bidni
Sample 52	Malta	2016	Bidni
Sample 53	Malta	2016	Carolea
Sample 54	Malta	2016	Frantoio
Sample 55	Malta	2016	Frantoio
Sample 56	Malta	2016	Frantoio
Sample 57	Malta	2016	Pendolino
Sample 58	Malta	2016	Picholine
Sample 59	Malta	2016	Carolea/Frantoio
Sample 60	Malta	2016	Carolea/Frantoio
Sample 61	Malta	2015	Mixed Local Coupage
Sample 62	Malta	2015	Mixed Local Coupage
Sample 63	Malta	2016	Mixed Local Coupage
Sample 64	Malta	2016	Mixed Local Coupage
Sample 65	Malta	2016	Mixed Local Coupage

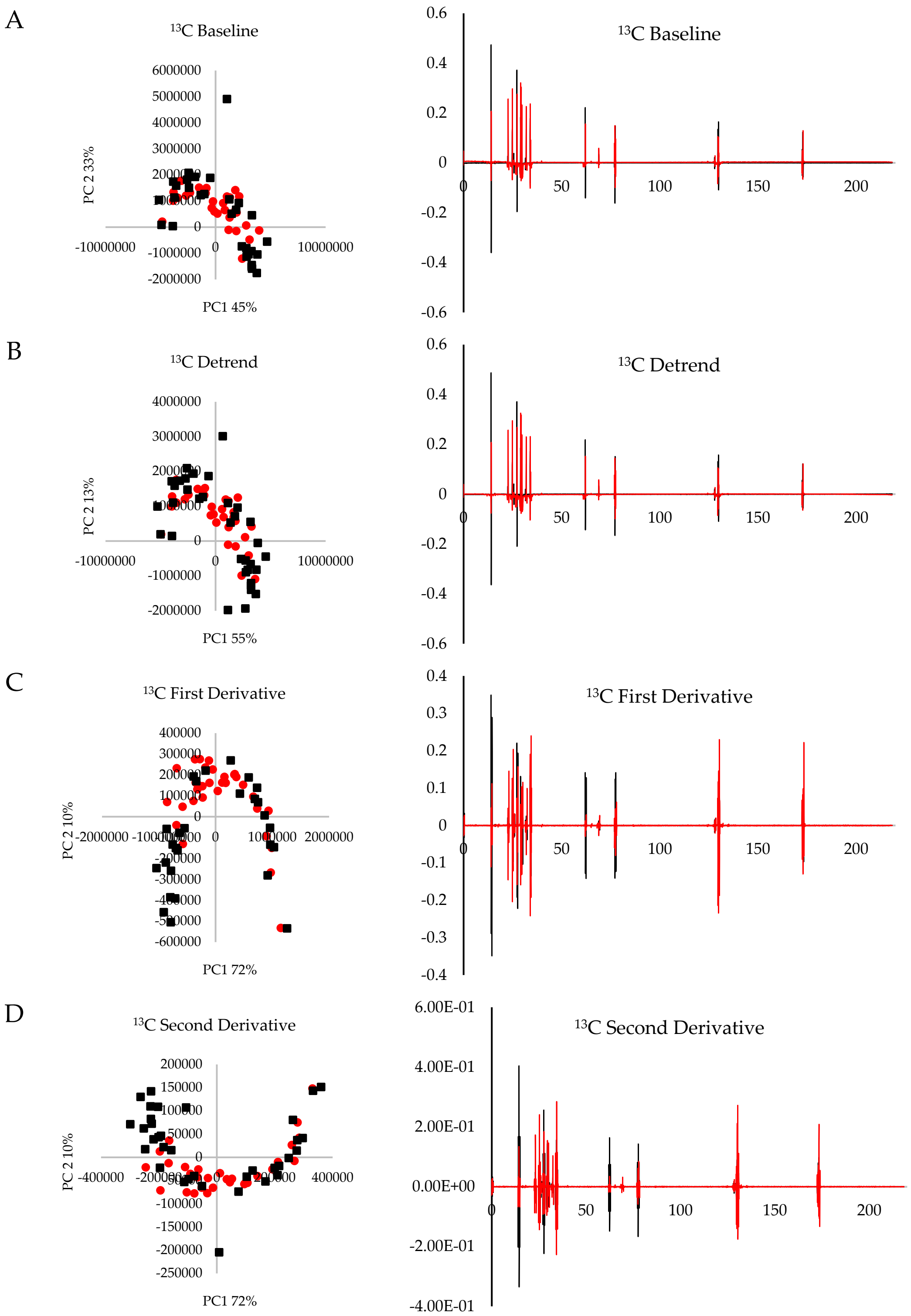

Figure S1. The principle component analysis (PCA) biplots (black boxes = Maltese red dots = non-Maltese) and loading plots for PC1 (black line) and PC2 (red line) for the (a) baseline corrected (b) Detrend (c) first derivative and (d) second derivative ^{13}C NMR spectra.

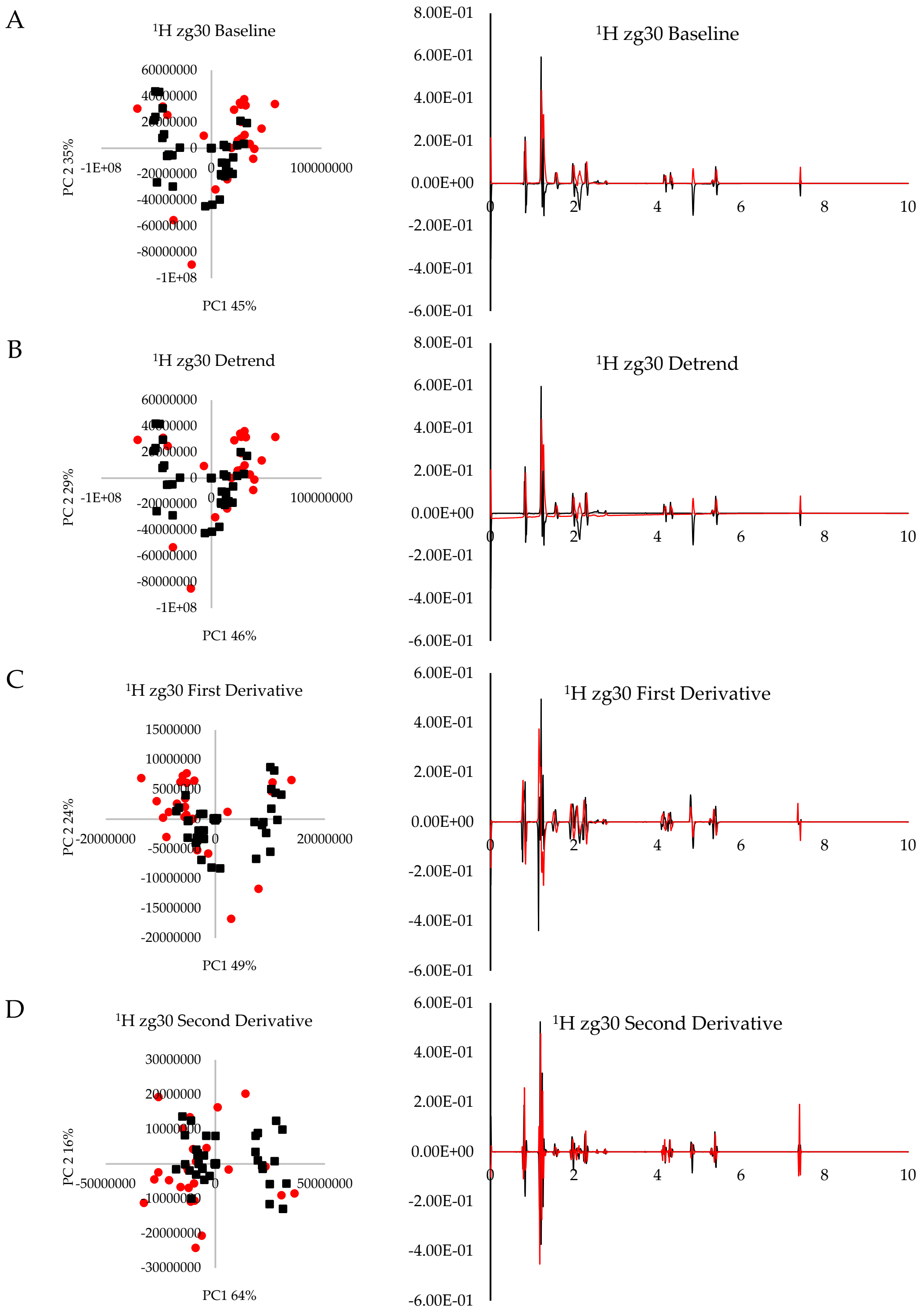

Figure S2. The principle component analysis (PCA) biplots (black boxes = Maltese red dots = non-Maltese) and loading plots for PC1 (black line) and PC2 (red line) for the (a) baseline corrected (b) Detrend (c) first derivative and (d) second derivative ¹H zg30 NMR spectra.

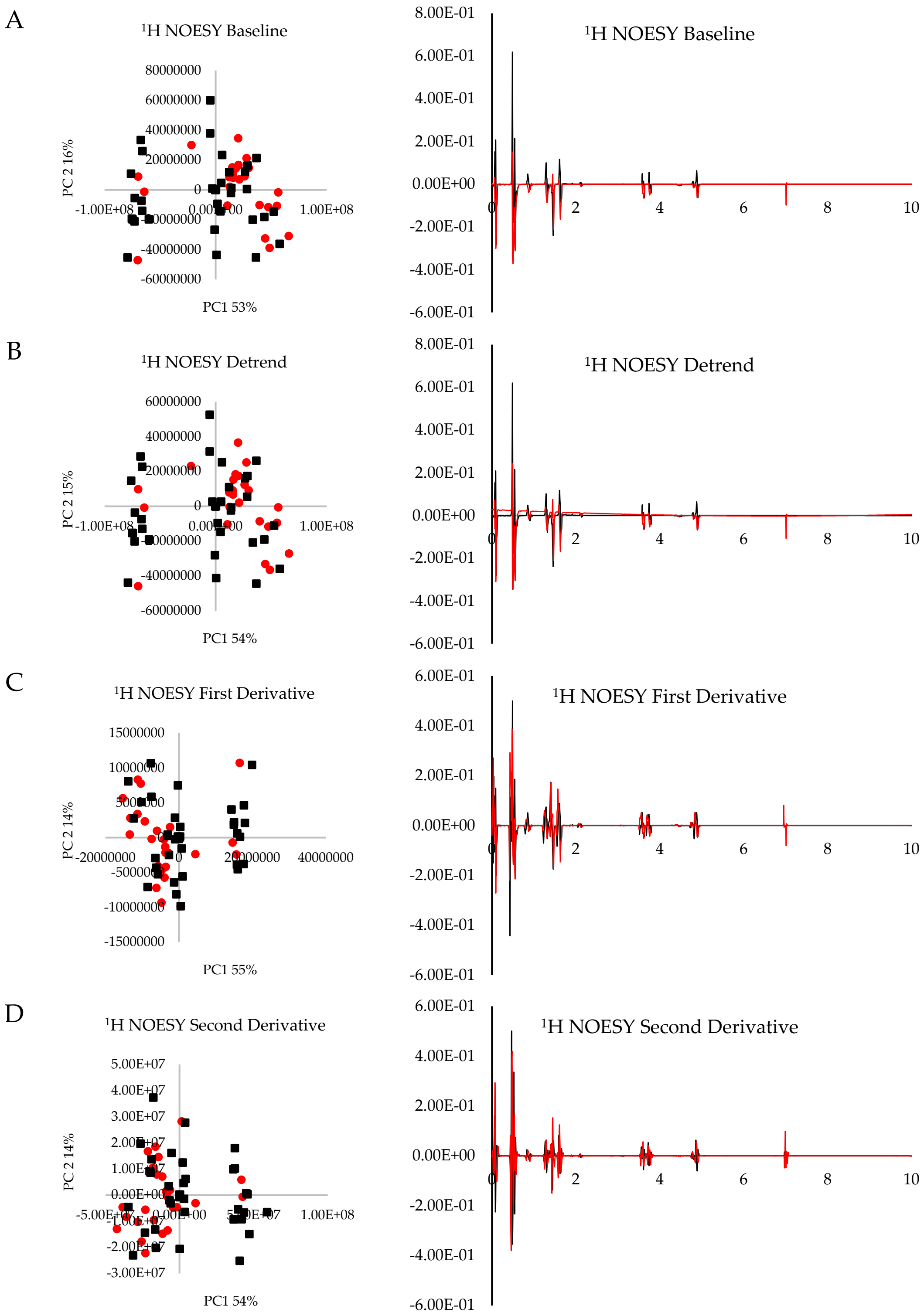

Figure S3. The principle component analysis (PCA) biplots (black boxes = Maltese red dots = non-Maltese) and loading plots for PC1 (black line) and PC2 (red line) for the (a) baseline corrected (b) Detrend (c) first derivative and (d) second derivative 1H NOESY NMR spectra.
